# Relationship between CT air trapping criteria and lung function in small airway impairment quantification

**DOI:** 10.1186/1471-2466-14-29

**Published:** 2014-02-28

**Authors:** Sébastien Bommart, Grégory Marin, Arnaud Bourdin, Nicolas Molinari, François Klein, Maurice Hayot, Isabelle Vachier, Pascal Chanez, Jacques Mercier, Hélène Vernhet-Kovacsik

**Affiliations:** 1 Radiology Department, CHU Montpellier, 371 avenue Doyen Gaston Giraud 34295, Montpellier cedex 05, France; 2 INSERM U 1046 Université Montpellier 1, Montpellier 2, Montpellier, France; 3 Statistics Department CHU Montpellier, Montpellier, France; 4 Respiratory Disease Department, CHU Montpellier, Montpellier, France; 5 Clinical Physiology Department, CHU Montpellier, Montpellier, France; 6 Respiratory Disease Department, CHU Marseille, Marseille, France

**Keywords:** Air trapping, Bronchiole, Single breath nitrogen test, Biomarker, Software-assisted CT quantification

## Abstract

**Background:**

Small airways are regarded as the elective anatomic site of obstruction in most chronic airway diseases. Expiratory computed tomography (CT) is increasingly used to assess obstruction at this level but there is no consensus regarding the best quantification method. We aimed to evaluate software-assisted CT quantification of air trapping for assessing small airway obstruction and determine which CT criteria better predict small airway obstruction on single breath nitrogen test (SBNT).

**Methods:**

Eighty-nine healthy volunteers age from 60 to 90 years old, underwent spirometrically-gated inspiratory (I) and expiratory (E) CT and pulmonary function tests (PFTs) using SBNT, performed on the same day. Air trapping was estimated using dedicated software measuring on inspiratory and expiratory CT low attenuation area (LAA) lung proportion and mean lung density (MLD). CT indexes were compared to SBNT results using the Spearman correlation coefficient and hierarchical dendrogram analysis. In addition, receiver operating characteristic (ROC) curve analysis was performed to determine the optimal CT air-trapping criterion.

**Results:**

43 of 89 subjects (48,3%) had dN2 value above the threshold defining small airway obstruction (i.e. 2.5% N2/l). Expiratory to inspiratory MLD ratio (r = 0.40) and LAA for the range −850 -1024 HU (r = 0.29) and for the range −850 -910 HU (r = 0.37) were positively correlated with SBNT results. E/I _MLD_ was the most suitable criterion for its expression. Expiratory to inspiratory MLD ratio (E/I _MLD_) showed the highest AUC value (0.733) for small airway obstruction assessment.

**Conclusion:**

Among all CT criteria, all correlating with small airway obstruction on SBNT, E/I _MLD_ was the most suitable criterion for its expression in asymptomatic subjects with mild small airway obstruction

**Trial registration:**

Registered at Clinicaltrials.gov, identifier: NCT01230879.

## Background

Chronic airway diseases are obstructive lung disorders occurring more and more frequently, therefore becoming a major public health burden worldwide [1]. Severity assessment and management of these diseases are defined according to clinical examination and routine pulmonary function tests (PFTs). However, these measurements are not strong enough to accurately discriminate patients regarding clinical outcomes [2]. Chronic obstructive pulmonary disease (COPD) and asthma are the most common causes of such chronic airflow limitation. From a pathophysiological point of view, small airways (defined from 2 mm in internal diameter and downward) are regarded as the underlying elective anatomic level of airway obstruction for both disorders [3,4], but are inadequately investigated using conventional PFTs, namely “the lung’s quiet zone” [5]. Specific structural changes at this level can be assessed using tools such as the single-breath or multiple-breath nitrogen washout test (SBNT, MBNT respectively) [6]. SBNT and MBNT are time consuming and the routine uses are not widely available owing to limited access to the equipment. Due to the fact that expiratory acquisition allows indirect evaluation of bronchiolar involvement [7], computed tomography (CT) has been increasingly put forward as an appropriate non-invasive tool for refinement in the classification and treatment monitoring of COPD and asthma [8-14]. The application of an increasingly wide range of technological tools also allows post-processing by segmentation software and thus quantifies air trapping objectively. Both a decrease in mean lung density and the percentage of low attenuation area on expiratory CT have been used in various study and correlated with disease severity in COPD and asthma [15-17]. Some authors have also suggested the role of paired expiratory to inspiratory ratio or difference as a small airway marker [18-20]. However, the choice of one of these criteria may affect the results and there is, therefore, a crucial need for standardization in CT air trapping expression.

This prospective study was designed firstly to explore the validity of air trapping software-assisted CT quantification for the assessment of small airway obstruction by using SBNT as standard of reference and, secondly, to assess which was the most accurate criterion.

## Methods

### Subjects and eligibility criteria

Between August 2009 and April 2012, we prospectively conducted a cross-sectional study on lung aging. This specific population was chosen to select a range of subtle small airway obstruction. Eligible participants were asymptomatic, non-smokers for at least 20 years with a cumulative history <10 pack-years of tobacco use and without a past history of lung disease. Subjects were asked to be in good mental and physical health as assessed by medical interview and physical activity using the Voorips score questionnaire [21]. All the final study participants had normal range spirometry, specifically regarding forced expiratory volume. They were informed of the aims of the study and gave their informed consent for both pulmonary function testing and chest CT.

This study received the approval of the local research ethics committee (CPP sud Méditerranée IV) and the agreement of the French Health Products Safety Agency (ANSM) before the start of the research.

### Scanning techniques

All CT examinations were performed using a 64–detector row CT scanner (LightSpeed VCT; GE Healthcare, Waukesha, USA) without the administration of contrast material. Patients were placed in the supine position. The entire chest from apex to posterior recesses was included in the cranio-caudal direction according to the following protocol: tube voltage: 120 kV, automatic tube current modulation with maximal current limited to 300 mAs, collimation: 64 × 0.6 mm, increment: 0.9, reconstructed slice thickness: 1.25 mm, tube rotation: 0.5 s, acquisition field of view ranged from 320 to 380 mm depending on the patient’s body habitus. Each chest CT examination was reconstructed using a standard filter.

Images were acquired using spirometric gating (WinspiroPRO; Medical International Research Waukesha, USA) to monitor lung volumes and determine respiratory endpoints. Patients were trained to breathe into a mouthpiece connected to the handheld spirometer after calibration of the spirometer according to manufacturer specifications. Breath-hold was obtained at least 3 times in inspiration and expiration using the spirometric equipment just before CT acquisition in supine position. Image sets were then acquired during single-breath-hold helical scanning between 90 and 100% of previously recorded slow vital capacity in supine position. Four additional slices were acquired in the same conditions at end expiration: one through the upper lobes, one through the tracheal carina and two between the tracheal carina and the diaphragm. All DICOM images were anonymously archived and transferred to a dedicated computer for post-treatment.

### Quantitative assessment of air trapping by CT

De-identified data was processed independently and blindly by a senior radiologist with 8 years of experience interpreting chest CT images (S.B.) and also by a chest radiology fellow (F.K.). Lung parenchyma was automatically isolated from the chest wall, mediastinum and air contained within the segmental bronchi (Additional file 1: Figure S1) and then analyzed using threshold techniques and histogram computation in order to quantify lung air trapping objectively and to provide reproducible data with the use of commercially available software (Myrian, Intrasense, Montpellier, France). Measurements were done for the four levels described above combining the values found for both sides at each level. Air trapping was defined in expiratory CT as lung regions that failed to increase in attenuation or decrease in volume in a normal fashion compared with findings on the initial inspiratory images [22]. Given the absence of a single, validated method of CT air trapping quantification, the inspiratory and expiratory chest CT scans were scored in 3 ways, most frequently used in the literature [23].

The percentage of lung parenchyma that fell within a range of low attenuating area (LAA%) was considered representative of air trapping: voxels between −850 and −1024 HU, called exp −850-1024 on expiratory slices were isolated. A second threshold was also applied between −850 and −910 HU (exp −850-910) to avoid low-density values due to emphysematous or cystic lesions. Finally, mean lung density was automatically measured.

Expiratory to inspiratory ratio and the difference between these values were calculated. 4 expiratory images were matched visually by using anatomical landmarks with 4 inspiratory images with the same slice thickness from the inspiratory images. E/I, E-I and (E-I)/I were expressed using the two thresholds described above for LAA% and for MLD.

### Single breath nitrogen test (SBNT)

SBNT was performed the same day, just before CT scanning. This test was used to look specifically for small airway abnormalities. This test was performed as previously reported by Bourdin and colleagues [24], using the Vmax apparatus (Vmax; Sensomedics, Yorba Linda, CA, USA) under the supervision of an expert in clinical physiology (M.H.), blinded to the CT results. Subjects were asked to breathe slowly and deeply to reach residual volume. They were then asked to inhale as deeply as possible to obtain total lung volume capacity at which point the breathing valve started the delivery of 100% O2 gas. Subjects were then asked to exhale immediately at a 0.3–0.5 l/s flow giving visual feedback on the computer screen to allow for the procedure to be followed correctly. The fractional expiratory nitrogen concentration was plotted against the expired volume. This helped determine the closing volume and the slope of this relationship when it reached the plateau known as the phase III nitrogen slope (delta N2 or dN2). See the Additional file 2: Figure S2 for the single breath nitrogen washout test curve.

### Statistical analysis

Quantitative variables were expressed as means or medians and compared using the Student t test or the Wilcoxon test. Qualitative variables were expressed as numbers (percentages) and compared using the Chi 2 test or the Fisher test as appropriate.

Non-parametric Spearman test was carried out to assess the relationship between functional and CT parameters. Distance and similarity between dN2 and CT air trapping criteria were then analyzed using dendrogram analysis based on the agglomerative hierarchical cluster tree. The best CT parameter was determined using receiver operating characteristic (ROC) curve analysis using standard dN2 cut-off = 2.5% N2/l. To test the diagnostic consistency of air trapping quantification for the two readers, inter-observer agreement was calculated using intra-class correlation coefficient [25]. A p-value of ≤ 0.05 was considered statistically significant. The statistical analysis was performed using statistical software SAS 9.3 (SAS Institute; Cary, NC), R 2.14.1 and SPSS 17.0 for Windows (SPSS, Chicago, Ill).

## Results

Among the 101 eligible subjects, a total of 89 performed adequate spirometric maneuvers for both PFTs and CT acquisitions. Software-assisted inspiratory and expiratory lung segmentation was achievable for all these subjects. Description of the study population is provided in Table 1.

**Table 1:** Demographic and spirometric characteristics of study subjects

**Variables**	**Mean**	**Std**
Age (y)	72,03	8,08
Height (cm)	162,14	8,93
Weight (kg)	67,05	11,54
BMI (Kg/m^2^)	25,53	3,49
FVC (l)	3,28	0,89
FVC (% predicted)	123,10	20,86
FEV1 (l)	2,37	0,64
FEV1 (% predicted)	89,31	17,02
FEV1/FVC	72,69	6,82
MEF (l)	2,84	1,14
MEF (% predicted)	110,09	40,98
TLC (l)	5,85	1,17
TLC (% predicted)	110,95	15,21
RV (l)	2,58	0,61
RV (% predicted)	117,13	26,21
FRC (l)	3,28	0,73
FRC (% predicted)	111,87	22,64
CV (l)	2,91	0,80
dN2 (% slope)	3,02	2,24

Note. Data are means ± standard deviations for quantitative variables.Percentages are expressed as a ratio of measured to predicted values. *FVC* = forced vital capacity, *FEV1* = forced expiratory volume in 1 second, *MEF* = mid forced expiratory flow, *TLC* = total lung capacity, *RV* = residual volume, *FRC* = functional residual capacity, *CV* = closing volume, *dN2* = phase III nitrogen slope.

### Intra-class correlation coefficient values

Raw data quantitative air trapping measures were assessed using MLD and LAA% having attenuation below −850 HU or between −850 and −910 HU. The match between the two observers yielded good to excellent inter-reader reliability for each (Additional file 3: Table S1). The lowest inter-class correlation coefficient between junior and senior radiologists was 0.91 for the fourth slice EXP_-850–910_.

### Spatial Heterogeneity

**Figure 1:**
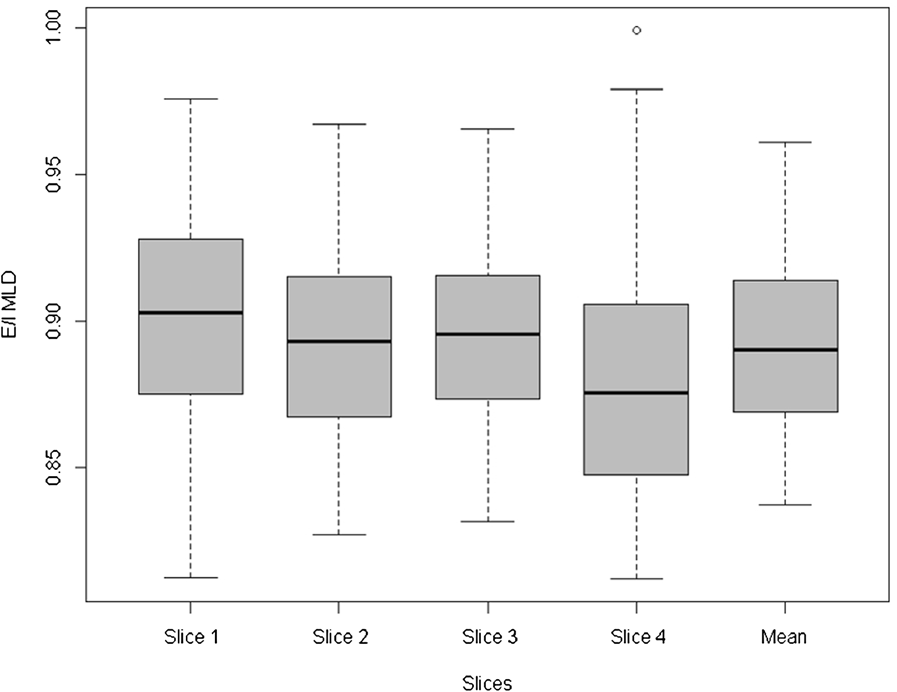
**Box plots of air trapping represented by E/I**_**MLD**_**in 4 levels and mean of E/I**_**MLD**_**.** Significant differences between slices were observed (p = 0.0002).

As shown in Figure 1, CT air trapping assessed by E/I _MLD_ ratio obtained at all four levels was statistically different (p = 0.02) but all the four expiratory mean lung density values remained significantly correlated with dN2 as demonstrated by the Spearman correlation coefficient (Additional file 4: Table S2).

### Comparison of CT indexes for small airway obstruction assessment

43 of 89 subjects (48,3%) had dN2 value above the cut-off (2.5% N2/l).

**Figure 2:**
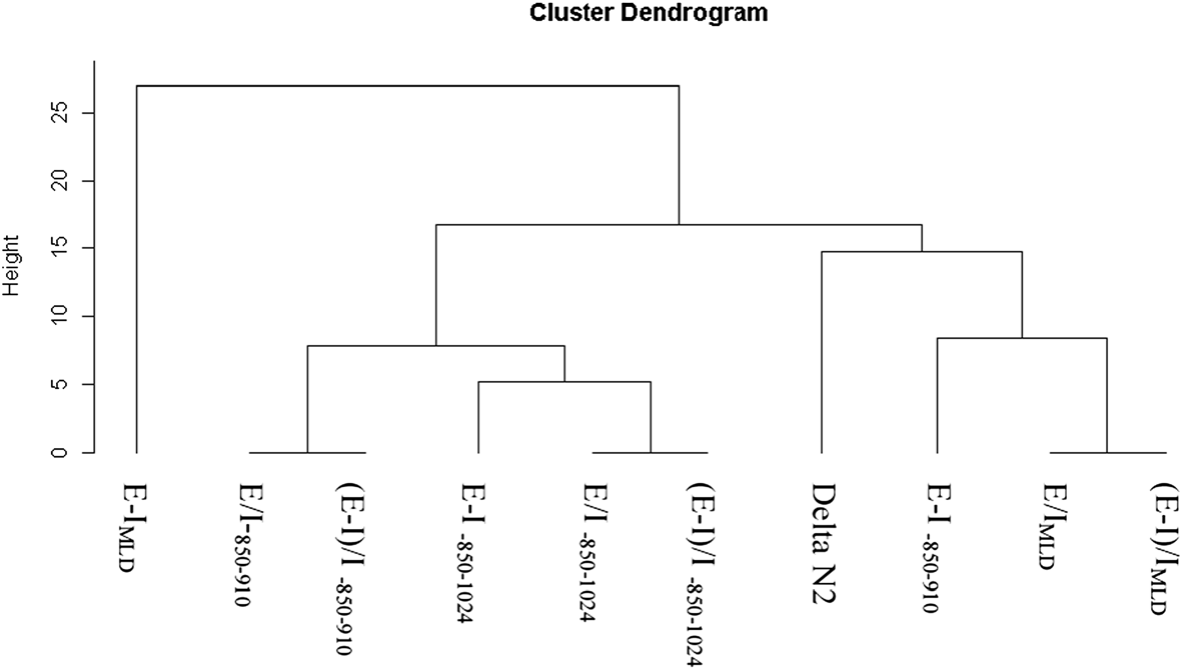
Hierarchical dendrogram analysis to evaluate distance and similarity between dN2 and CT air trapping criteria.

**Figure 3:**
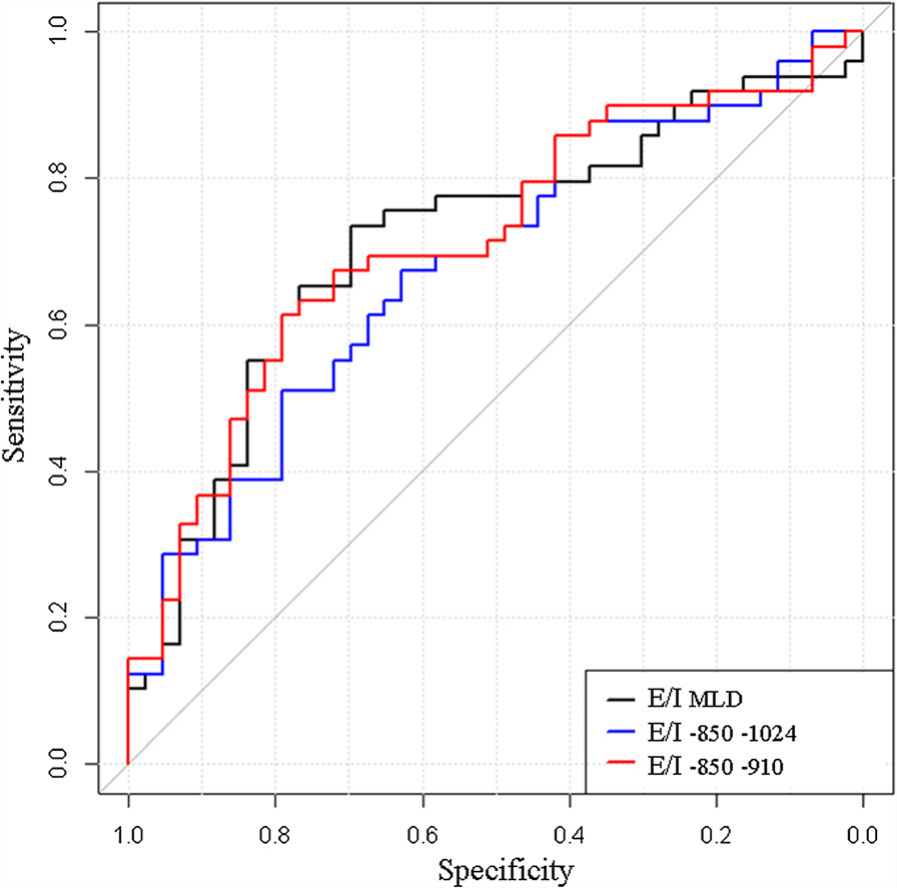
**Receiver-operating characteristic (ROC) curves show the diagnostic performance of E/I using dN2 as a functional test of airway obstruction (dN2 cut-off = 2.5% N2/l).** The best threshold is reported, alongside the sensitivity and specificity: E/I _MLD_ = 0.89 (0.70; 0.73) – E/I _-850–1024_ = 0.32 (0.63; 0.67) – E/I _-850–910_ = 0.29 (0.79; 0.61).

**Figure 4:**
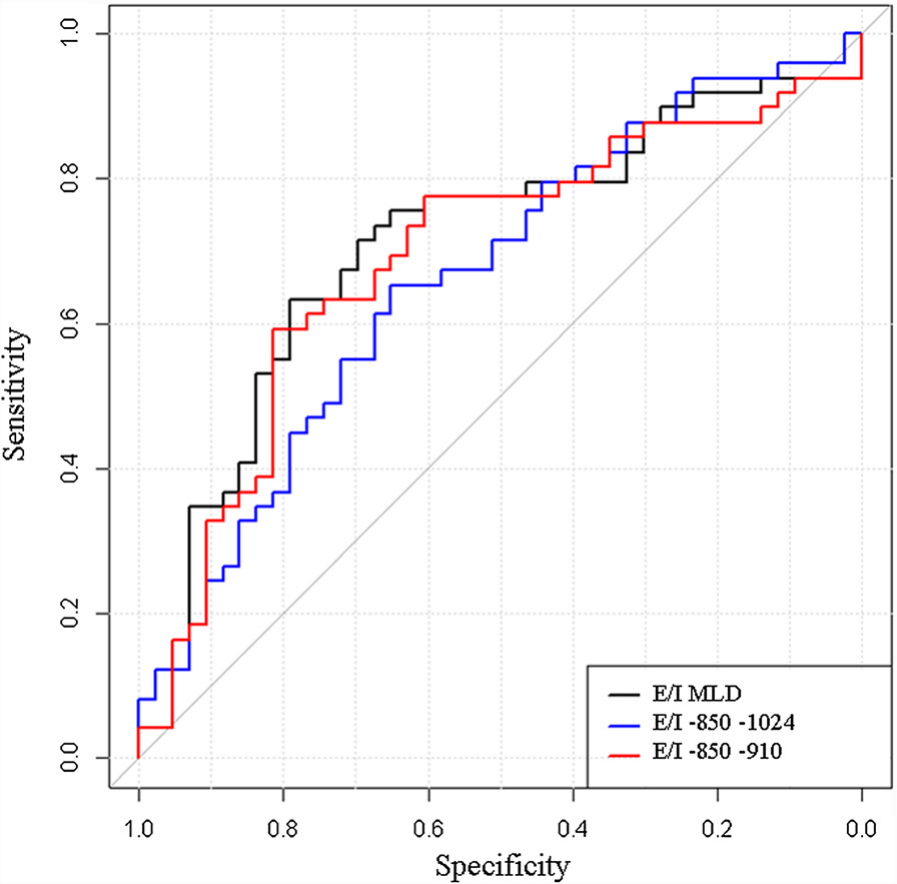
**Receiver-operating characteristic (ROC) curves show the diagnostic performance of E-I using dN2 as a functional test of airway obstruction (dN2 cut-off = 2.5% N2/l).** The best threshold is reported, alongside the sensitivity and specificity: E-I _MLD_ = 90.01 (0.79; 0.63) – E-I _-850–1024_ = −34.24 (0.65; 0.65) – E-I _-850–910_ = −47.23 (0.81; 0.59).

**Figure 5:**
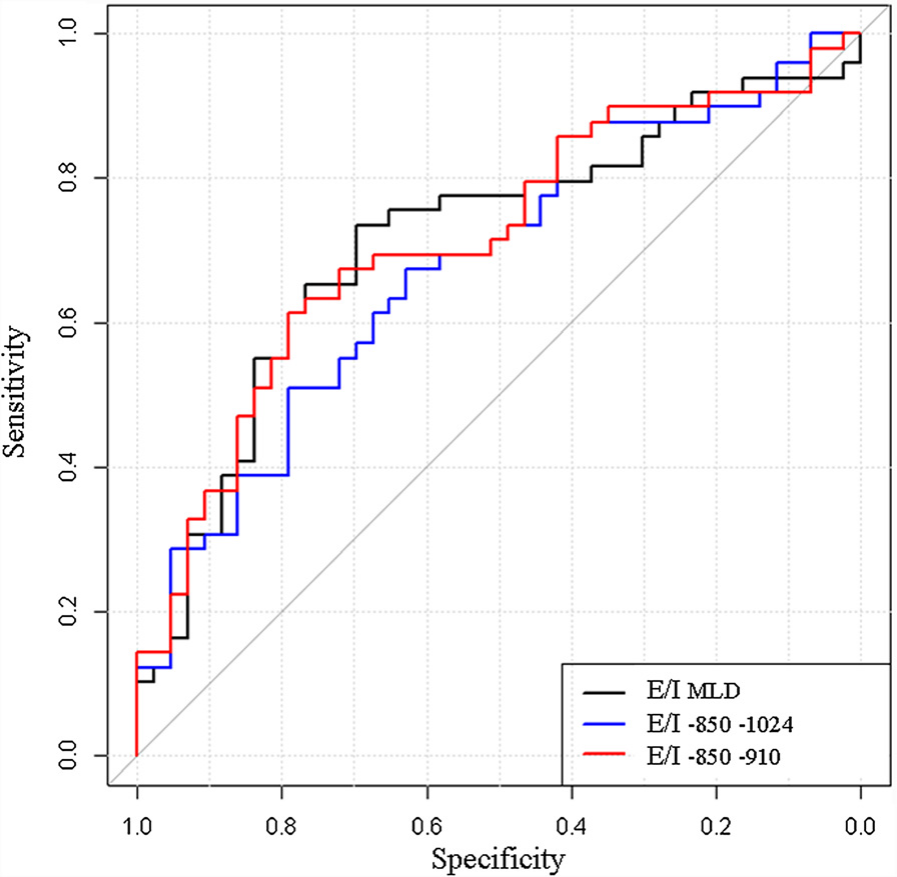
**Receiver-operating characteristic (ROC) curves show the diagnostic performance of (E-I)/I using dN2 as a functional test of airway obstruction (dN2 cut-off = 2.5% N2/l).** The best threshold is reported, alongside the sensitivity and specificity: (E-I)/I _MLD_ = −0.11 (0.70; 0.73) – (E-I)/I _-850–1024_ = −0.68 (0.63; 0.67) – (E-I)/I _-850–910_ = −0.70 (0.79; 0.61).

All CT indexes (E/I, (E-I)/I and E-I LAA% or MLD correlated with small airway obstruction, as assessed by dN2 (Additional file 4: Table S2). Figure 2 shows the connection between dN2 and the CT criteria generated by hierarchical dendrogram analysis. The cluster located the furthest from dN2 was E-I_MLD_. The three branches the most closely related to dN2 in the cluster dendrogram (E/I_MLD_, (E-I)/I_MLD_, E-I_-850–910_) were then included for further ROC curve analysis to determine the criteria that yielded the highest combined sensitivity and specificity and thus the highest diagnostic value. The resulting ROC curves are shown Figures 3, 4 and 5. E/I_MLD_ provided the best area under the curve (AUC) value (0.714 confidence interval: 0.606, 0.822) using dN2 as a reference. Similar results were obtained for (E-I)/I. When analysis was performed on the second slice individually, AUC for E/I and (E-I)/I was slightly higher (0.733; 0.630 – 0.836).

## Discussion

This study reports a clear relationship between spirometrically gated CT air trapping quantification and physiological measurements of the small airways using SBNT. The strength of the CT quantification technique is its capacity to express small airway obstruction reliably using standard, commercially available, software. Indeed, software-assisted CT air trapping quantification was feasible in our study for all subjects who achieved adequate CT acquisitions with a high consistency between observers even for inspiratory images, matched visually by using anatomical landmarks. Expiratory to inspiratory ratio and the difference between these values were calculated to characterize CT air trapping in order to overcome error related to decrease in lung density due to alveolar enlargement likely associated with age [26-28]. E/I_MLD_ and (E-I)/I _MLD_ exhibited the best performance characteristics to assess small airway obstruction. O. Mets and colleagues recently pointed out that E/I_MLD_ was related to PFTs [29]. They showed a relationship between residual volume/Total lung volume capacity (RV/TLC) and E/I _MLD_ in a population of current and former heavy smokers in a lung cancer screening cohort. Nonetheless, RV/TLC reflects static lung hyperinflation and the value may be influenced either by the presence of small airway obstruction or by emphysema. Furthermore, discrimination is possible only in severe cases of hyperinflation. Only 38 of the 427 heavy smokers subjects presented abnormal RV/TLC in the Mets study. There is currently no functional test allowing perfect non-invasive assessment of small airways. Nonetheless inert gas washout tests such as SBNT provide information on ventilation inhomogeneity that allows sensitive detection of small airway obstruction even when RV/TLC or other standard spirometry values are normal [30-33]. Our sample population aged 60 and older and the selection criteria used offered a well-defined population of subjects with a range of small airway obstruction that may be attributed to normal ageing [34,35]. Furthermore, people included in this study achieved reproducible maneuvers that are a key point for SBNT validity [6].

The correlation found in our study between CT and SBNT showed that CT air trapping can detect even mild to moderate small airway obstruction as seen in our subjects who were all asymptomatic but nevertheless having physiological modifications. The aim was to validate CT air trapping quantification from a homogeneous model that could be further applied to various chronic obstruction situations such as COPD or asthma.

Our study has several potential limitations. First, we did not used whole expiratory volume also described for air trapping assessment but only a single set of four expiratory slices [19,36]. We did this because of the difficulty for subjects to sustain breath-hold at low lung volumes and furthermore in keeping with the recommendation made by the ethics committee to limit the radiation dose as well as the “As Low As Reasonably Achievable” principle. We cannot know from this study whether analysis on the whole lung volume would have improved the correlations, nonetheless our results showed similar significant air trapping heterogeneity between the upper, middle, and lower regions to that found by Bankier and colleagues in their work on a lung transplant population [37]. Regardless of slice level, each remained significantly associated with dN2. Even if AUC was slightly higher for E/I _MLD_ in slice 2 (i.e. through the carina), we did not recommend the use of only one expiratory slice as its index could not be applied to pathologies such as asthma, which have been proven to have widely heterogeneous spread [38].

Second, although use of a spirometer to control lung volume during acquisition is not standard practice, in this study it provided proof of inspiration and expiration volume.

Another potential limitation is the lack of emphysematous lesions in our population. The elective site of measurement should be adapted in COPD patients with such lesions as stated by Matsuoka [39].

## Conclusions

Our study demonstrated that in asymptomatic subjects with small airway obstruction, software-assisted CT air trapping quantification correlated with SBNT. E/I and (E-I)/I _MLD_ are equally representative of small airway obstruction and could be used as a small airway obstruction biomarker.

## Abbreviations

COPD: Chronic obstructive pulmonary disease; CT: Computed tomography; delta N2: Phase III nitrogen slope; LAA: Low attenuation area; MBNT: Multi breath nitrogen test; MLD: Mean lung density; PFT: Pulmonary function test; ROC: Receiver operating characteristic; RV: Residual volume; SBNT: Single breath nitrogen test; TLC: Total lung volume capacity.

## Competing interests

The study was funded by a grant from the French Ministry of Health (Programme Hospitalier de Recherche Clinique 2008). No potential conflicts of interest relevant to this article were reported for any authors.

## Authors’ contribution

SB and HK were responsible for the concept and design of the study. SB drafted and wrote the paper. AB participates in the study design and helped to draft the manuscript. GM and NM helped to perform statistical analysis. All authors contributed to the clinical data collection, data analysis and review the manuscript. All authors read and approved the final manuscript.

## Pre-publication history

The pre-publication history for this paper can be accessed here:

http://www.biomedcentral.com/1471-2466/14/29/prepub

## Supplementary Material

Additional file 1: Figure S1 Automated quantification of air trapping using segmentation software obtained in a 64-year old woman. Axial expiratory images. Color code: Blue for total lung parenchyma (areas with attenuation of -500 to -1024 HU). Red for attenuation between -850 and -910 HU and green for attenuation less than -910 HU. Click here for file

Additional file 2: Figure S2 Schematic representation of single breath nitrogen test. Click here for file

Additional file 3: Table S1 Comparison of the inter-observer agreement (Intra-Class correlation*). Click here for file

Additional file 4: Table S2 Spearman correlations between Delta N2 and (expiratory/inspiratory) values, for the four slices. Click here for file
