# Supplementary material for: Relationship between CT air trapping criteria and lung function in small airway impairment quantification
Source: BMC Pulm Med. 2014 Feb 28;14:29. doi: 10.1186/1471-2466-14-29 (PMC4015710; doi:10.1186/1471-2466-14-29)

Online Supplementary Figure Automated quantification of air trapping using segmentation software obtained in a 64-year old woman.

Axial expiratory images. Color code: Blue for total lung parenchyma (areas with attenuation of -500 to -1024 HU). Red for attenuation between -850 and -910 HU and green for attenuation less than -910 HU.

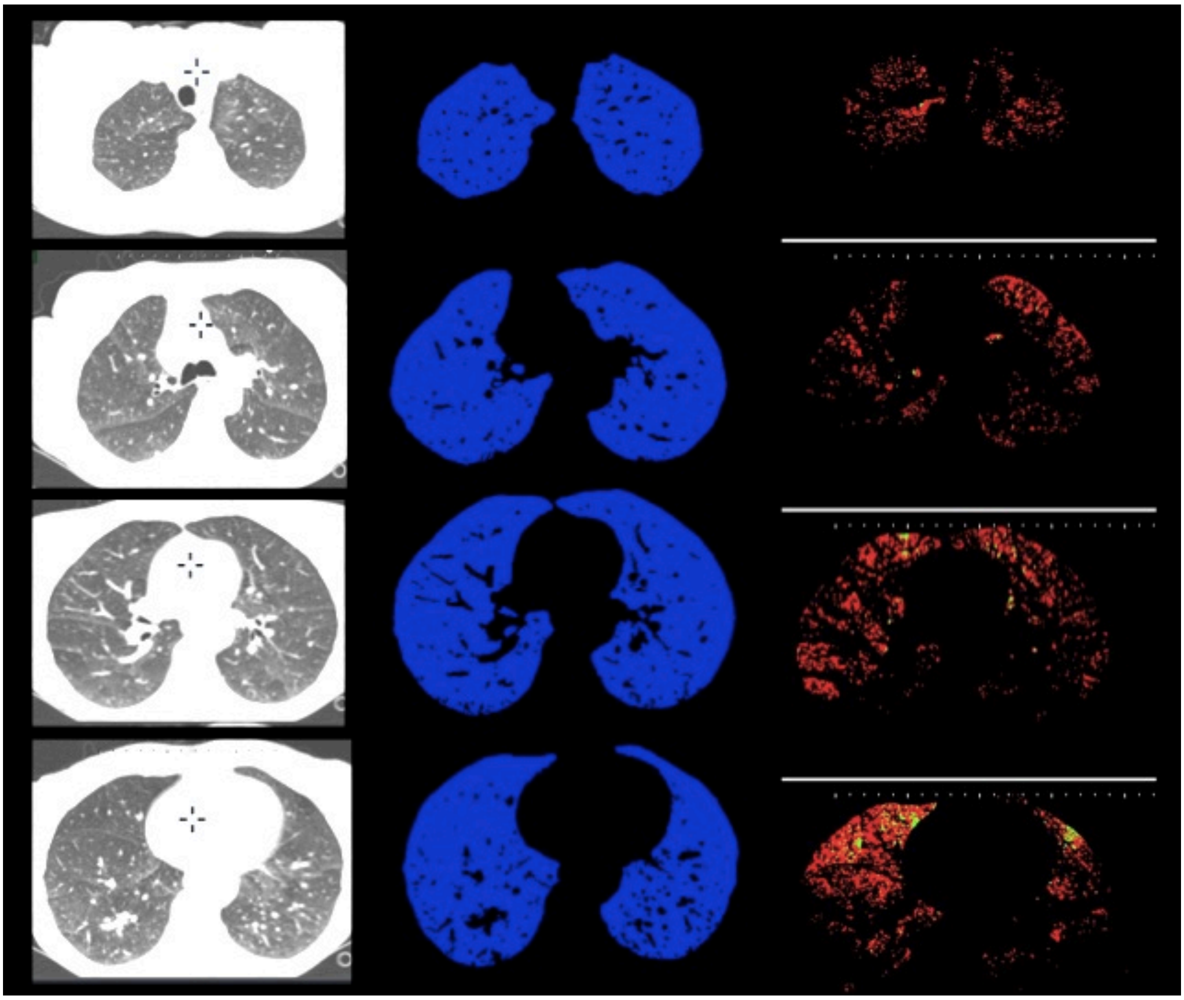

Supplement: Additional file 1: Figure S1 — Automated quantification of air trapping using segmentation software obtained in a 64-year old woman. Axial expiratory images. Color code: Blue for total lung parenchyma (areas with attenuation of -500 to -1024 HU). Red for attenuation between -850 and -910 HU and green for attenuation less than -910 HU. [file 1471-2466-14-29-S1.pdf]
