# Supplementary material for: Relationship between CT air trapping criteria and lung function in small airway impairment quantification
Source: BMC Pulm Med. 2014 Feb 28;14:29. doi: 10.1186/1471-2466-14-29 (PMC4015710; doi:10.1186/1471-2466-14-29)
Supplement: Additional file 2: Figure S2 — Schematic representation of single breath nitrogen test. [file 1471-2466-14-29-S2.pdf]

Schematic representation of single breath nitrogen test

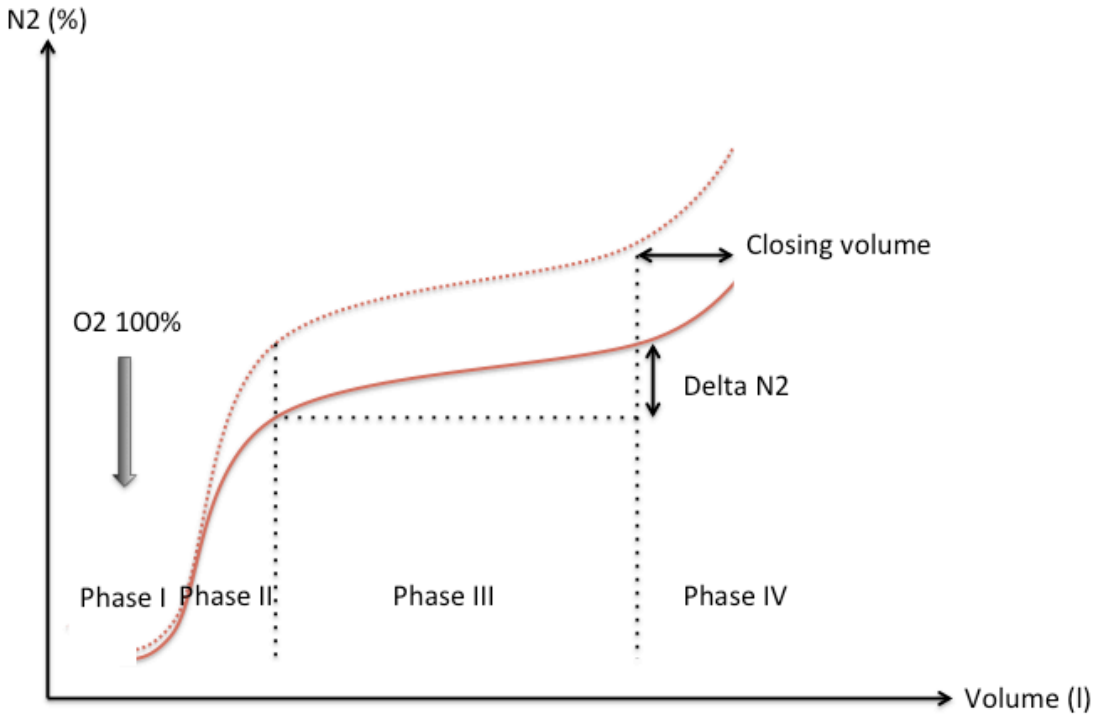

The nitrogen gas (N2) concentration is plotted against expired volume following a breath of 100% oxygen. The phase I and II correspond to the dead space and the bronchial phase. The phase III characterizes the alveolar plateau. The last part of the forced expiration (phase IV) is marked by a fast increase in the concentration of expired nitrogen. This change reflects the closing volume (i.e. small airway closure).
In this graph, the trace is obtained in a normal subject (solid line) and in a subject with small airway obstruction (dashed line).
