# Supplementary material for: Relationship between CT air trapping criteria and lung function in small airway impairment quantification
Source: BMC Pulm Med. 2014 Feb 28;14:29. doi: 10.1186/1471-2466-14-29 (PMC4015710; doi:10.1186/1471-2466-14-29)
Supplement: Additional file 3: Table S1 — Comparison of the inter-observer agreement (Intra-Class correlation*). [file 1471-2466-14-29-S3.pdf]

Online Supplementary Table 1 Comparison of the inter-observer agreement (Intra-class correlation*)

CT parameters	ICC*	Confidence interval	
$EXP1_{MLD}$	1	.	.
$EXP1_{-850-910}$	1	.	.
$EXP1_{-850-1024}$	1	.	.
$EXP2_{MLD}$	0.95	0.92	0.97
$EXP2_{-850-910}$	0.99	0.98	0.99
$EXP2_{-850-1024}$	0.99	0.98	0.99
$EXP3_{MLD}$	1	.	.
$EXP3_{-850-910}$	1	.	.
$EXP3_{-850-1024}$	1	.	.
$EXP4_{MLD}$	1	.	.
$EXP4_{-850-910}$	0.91	0.89	0.92
$EXP4_{-850-1024}$	0.96	0.95	0.98
$INSP1_{MLD}$	0.90	0.88	0.95
$INSP1_{-850-910}$	0.97	0.94	0.99
$INSP1_{-850-1024}$	0.92	0.88	0.95
$INSP2_{MLD}$	0.97	0.96	0.99
$INSP2_{-850-910}$	0.95	0.93	0.97
$INSP2_{-850-1024}$	1	.	.
$INSP3_{MLD}$	0.99	0.98	0.99
$INSP3_{-850-910}$	0.99	0.98	0.99
$INSP3_{-850-1024}$	0.99	0.98	0.99
$INSP4_{MLD}$	0.99	0.98	0.99
$INSP4_{-850-910}$	0.98	0.98	0.99
$INSP4_{-850-1024}$	0.99	0.98	0.99

ICC was calculated on the raw variables measured by the 2 observers.

Exp $_{-850-910}$: Lung voxels having attenuation between -850 and -910 HU on expiratory slices at each level. Exp $_{-850-1024}$: Lung voxels having attenuation between -850 and -1024 HU on expiratory slices at each level. INSP $_{-850-910}$: Lung voxels having attenuation between -850 and -910 HU on inspiratory images. INSP $_{-850-1024}$: Lung voxels having attenuation between -850 and -1024 on inspiration set matched images. MLD = Mean lung density.
