# Supplementary material for: Relationship between CT air trapping criteria and lung function in small airway impairment quantification
Source: BMC Pulm Med. 2014 Feb 28;14:29. doi: 10.1186/1471-2466-14-29 (PMC4015710; doi:10.1186/1471-2466-14-29)
Supplement: Additional file 4: Table S2 — Spearman correlations between Delta N2 and (expiratory/inspiratory) values, for the four slices. [file 1471-2466-14-29-S4.pdf]

Online Supplementary Table 2 Spearman correlations between Delta N2 and (expiratory/inspiratory) values, for the four slices.

	Delta N2	
	value	P
**E/I_{MLD}1**	0.39444	**0.0001**
**$E/I_{-850-1024}$ 1**	0.31606	**0.0021**
**$E/I_{-850-910}$ 1**	0.35737	**0.0005**
**E/I_{MLD} 2**	0.45451	**<.0001**
**$E/I_{-850-1024}$ 2**	0.30639	**0.0030**
**$E/I_{-850-910}$ 2**	0.37390	**0.0002**
**E/I_{MLD} 3**	0.38598	**0.0001**
**$E/I_{-850-1024}$ 3**	0.23977	**0.0213**
**$E/I_{-850-910}$ 3**	0.34211	**0.0008**
**E/I_{MLD} 4**	0.29793	**0.0039**
**$E/I_{-850-1024}$ 4**	0.18236	**0.0819**
**$E/I_{-850-910}$ 4**	0.29647	**0.0041**
**E/I_{MLD} MEAN**	0.40075	**<.0001**
**$E/I_{-850-1024}$ MEAN**	0.29556	**0.0042**
**$E/I_{-850-910}$ MEAN**	0.37355	**0.0002**
**$(E-I)/I_{MLD}$ 1**	0.39444	**0.0001**
**$(E-I)/I_{-850-1024}$ 1**	0.31606	**0.0021**
**$(E-I)/I_{-850-910}$ 1**	0.35737	**0.0005**
**$(E-I)/I_{MLD}$ 2**	0.45451	**<.0001**
**$(E-I)/I_{-850-1024}$ 2**	0.30639	**0.0030**
**$(E-I)/I_{-850-910}$ 2**	0.37390	**0.0002**
**$(E-I)/I_{MLD}$ 3**	0.38598	**0.0001**
**$(E-I)/I_{-850-1024}$ 3**	0.23977	**0.0213**
**$(E-I)/I_{-850-910}$ 3**	0.34211	**0.0008**
**$(E-I)/I_{MLD}$ 4**	0.29793	**0.0039**
**$(E-I)/I_{-850-1024}$ 4**	0.18236	**0.0819**
**$(E-I)/I_{-850-910}$ 4**	0.29647	**0.0041**
**$(E-I)/I_{MLD}$ MEAN**	0.40075	**<.0001**
**$(E-I)/I_{-850-1024}$ MEAN**	0.29556	**0.0042**
**$(E-I)/I_{-850-910}$ MEAN**	0.37355	**0.0002**
